# The BRD4-SRPK2-SRSF2 signal modulates the splicing efficiency of ACSL3 pre-mRNA and influences erastin-induced ferroptosis in osteosarcoma cells

**DOI:** 10.1038/s41419-023-06273-2

**Published:** 2023-11-23

**Authors:** Shun-Hong Luo, Jia-Ming Tian, Yi Chu, Hong-Yi Zhu, Jiang-Dong Ni, Jun Huang

**Affiliations:** 1 Department of Orthopedics, The Second Xiangya Hospital, Central South University, Changsha, Hunan China; 2 Department of Gastroenterology, The Second Xiangya Hospital, Central South University, Changsha, Hunan China

**Keywords:** Bone cancer, Cell death

## Abstract

Lipid metabolism is the key to ferroptosis susceptibility. However, little is known about the underlying mechanisms in osteosarcoma cells. Functional restriction of bromodomain-containing protein 4 (BRD4) reduced the susceptibility to erastin-induced ferroptosis of osteosarcoma cells both in vitro and in vivo. Mechanically, BRD4 controls the splicing efficiency of the RNA precursor (pre-mACSL3) of ACSL3 (ACSL3) by recruiting serinerich/threonine protein kinase 2 (SRPK2) to assemble the splicing catalytic platform. Moreover, the AMP-binding domain of ACSL3 significantly influences arachidonic acid synthesis and thus determines the susceptibility to erastin-induced ferroptosis. Overall, we found a BRD4-mediated pre-mACSL3 splicing influences erastin-induced ferroptosis by affecting arachidonic acid synthesis in osteosarcoma cells. Data in this study fills some of the gap in understanding the post-transcriptional regulatory mechanisms of ACSL3 and provides new insights into the mechanisms of lipid metabolism regulation and its effect on susceptibility to ferroptosis in osteosarcoma cells.

## Highlights

- Inhibition of BRD4 decreased the sensitivity of osteosarcoma cells to erastin-induced ferroptosis.
- BRD4 recruits SRPK2 to catalyze the splicing efficiency of SRSF2 on pre-mACSL3.
- The AMP-binding domain of ACSL3 is required for arachidonic acid anabolism and contributes to increased susceptibility of osteosarcoma cells to erastin-induced ferroptosis.

## Introduction

Ferroptosis is a programmed cell death process driven by iron-dependent lipid peroxidation [1]. Induction of ferroptosis is of great value in addressing malignancies that exhibit resistance to conventional treatment strategies or are characterized by therapeutic challenges [2]. Currently, nanoparticles have shown good effects against osteosarcoma cells through stimulating ferroptosis [3, 4], suggesting that inducing ferroptosis is a feasible approach in the treatment of osteosarcoma. But so far, little is known about the targets and mechanisms of ferroptosis in osteosarcoma [5].

Lipid metabolism is essential for the energy supply of osteosarcoma cells [6]. Unlike healthy individuals, patients with osteosarcoma often exhibit hyperactive lipid metabolism, so targeting this metabolic characteristic is considered one of the promising strategies against osteosarcoma [7]. Moreover, the susceptibility to ferroptosis is significantly affected by lipid metabolism, which may also provide an opportunity for precise treatment by allowing specific targeting of cancer cells with high metabolic levels to stimulate ferroptosis while preserving normal tissues [8]. According to reports, lipid metabolic enzymes such as stearoyl-CoA desaturase 1 (SCD1) [9] and long-chain fatty acyl-CoA synthetase 4 (ACSL4) [10] are involved in the regulation of ferroptosis. However, the current understanding is still incomplete, and further investigation of molecular targets linking lipid metabolism and ferroptosis is warranted.

Long-chain acyl-CoA synthase 3 (ACSL3) is a key regulator of lipid homeostasis [11–13]. Recent studies have demonstrated that ACSL3 exhibits a strong affinity for saturated fatty acids ranging from 8 to 22 carbons, as well as unsaturated fatty acids with 16–20 carbons such as arachidonic acid [13]. Furthermore, upregulation of ACSL3 leads to enhanced cellular uptake of exogenous fatty acids, subsequently promoting their conversion into β-oxidation for fatty acid synthesis [14]. Studies have revealed that ACSL3 exhibits a series of alternative splicing isoforms, with the only known variant located at the 5ʹ-untranslated region (5ʹ-UTR) of ACSL3 mRNA precursor (pre-mRNA), resulting in differential subcellular localization of ACSL3 onto different organelle membranes [12]. And working backward the potential isoforms from the known ACSL3 amino acid (aa) sequence on the *Uniprot* website (https://www.uniprot.org/), we found that the complete isoform has a total of 720 aa, including a type III transmembrane domain signal anchor (21–44 aa) in the N-terminus, an AMP-binding domain (113–587 aa) in the middle, and disorder regions in the rest. The observation that the AMP binding domain of ACSL3 undergoes frequent alterations or deletions, as well as exon skipping events, as reported by the *ExonskipDB* database (https://ccsm.uth.edu/ExonSkipDB/), has captured our attention. Consequently, we propose the hypothesis that post-transcriptional splicing of pre-mACSL3 could potentially impact its role as a long-chain fatty acid CoA ligase, resulting in a distinct response to AMP-dependent lipid metabolic signals.

Bromodomain-containing protein 4 (BRD4), a member of the bromodomain and extra-terminal family (BET), has attracted great attention from academia and the pharmaceutical industry due to its great potential as a new target for a variety of cancers [15]. Research shows key roles of BRD4 in lipid accumulation-related diseases [16, 17], but its biological mechanisms have not been fully elucidated. Presently, research findings have provided evidence of the direct or indirect engagement of BRD4 in the regulation of pre-mRNA splicing. For instance, Amit K. Singh et al. found that BRD4 directly interacts with fused-in sarcoma protein (FUS), heterogeneous nuclear ribonucleoprotein M (HnRNPM), and heterogeneous nuclear ribonucleoprotein L (HnRNPL), implying its involvement as a component of the splicing machinery in the regulation of splicing [18]. Additionally, Michal R. Schweiger et al. identified BRD4 as a significant regulator of splicing in response to heat stress [19]. However, the precise mechanism by which BRD4 is involved in splicing regulation remains incomplete and requires further investigation.

A study on lung cancer has indicated that the absence of ACSL3 leads to intracellular ATP depletion [20], which aligns with our previous observations in cells following BRD4 inhibition [21]. Furthermore, whole-body knockout of ACSL3 in mice does not result in lethality or any overt dysfunction, highlighting the vulnerability of cancer cells to targeting ACSL3 [20] and prompting us to investigate whether the splicing process of pre-mACSL3 is associated with BRD4, thereby acting as a downstream effector influencing ferroptosis.

In this study, we first determined the contribution of BRD4 and ACSL3 to erastin- induced ferroptosis in osteosarcoma cells, and then elucidated the effects and regulation mechanisms of BRD4 on pre-mACSL3 splicing and expression. The present study provides new insights into the mechanisms of lipid metabolism regulation in osteosarcoma cells and its effect on susceptibility to ferroptosis.

## Materials and methods

### Cell culture and treatment

SaoS2 and U2-OS cells with accurate STR identification were purchased from Fenghui Biotechnology (Changsha, China) and cultured in high glucose DMEM (Gibco, Cat. NO. 11965–092) supplemented with 10% fetal bovine serum (FBS) (Invitrogen, Cat.NO. 16000–044) at 37 °C, 5% CO_2_ in humidified air. For plasmid transfection or cell cycle synchronization, cells were serum-starved with 0.5% FBS for 12 h.

### Real-time fluorescent quantitative PCR (RT-qPCR)

Total RNA was isolated from osteosarcoma cells using TRIzol Reagent (Invitrogen, Cat. NO. 15596-026), followed by reverse transcription using a cDNA reverse transcription kit (Takara, Cat. NO. RR037A). Subsequently, RT-qPCR was conducted using the SYBR green PCR master mix (Takara, Cat. NO. RR430B). GAPDH was used as an internal control for normalisation. Finally, the relative expression of the target transcript was determined using the 2^-ΔΔCT^ method. The following primers were designed for RT-qPCR amplification and sequencing:

BRD4 forward: 5ʹ-GTGGTGCACATCATCCAGTC-3ʹ and reverse: 5ʹ-CCGACTCTGAGGACGAGAAG-3ʹ;

ACSL3 forward: 5ʹ-GTTTGGCTTCAGTATTATACCCT-3ʹ and reverse: 5ʹ-TTCGAGTTCAGTTAGTTCCT-3ʹ;

ACSL1 forward: 5ʹ-CAAGCAAACACCACGCTGAA-3ʹ and reverse: 5ʹ-CAAGCAAACACCACGCTGAA-3ʹ;

ACSL4 forward: 5ʹ-ATACCTGGACTGGGACCGAA-3ʹ and reverse: 5ʹ-TGCTGGACTGGTCAGAGAGT3ʹ;

ACSL5 forward: 5ʹ-CACCCCAAAAGGCATTGGTG-3ʹ and reverse: 5ʹ-CACCCCAAAAGGCATTGGTG3ʹ;

ACSL6 forward: 5ʹ-TGACCTTCTTCCTCGTGTCG-3ʹ and reverse: 5ʹ-CCATACTCACGAGGGTGGTG3ʹ;

GAPDH forward: 5ʹ-CACCATCTTCCAGGAGCGAGA-3ʹ and reverse: 5ʹ-CATGACGAACATGGGGGCAT-3ʹ.

### Western blotting

Total protein lysates from osteosarcoma cells were separated by sodium dodecyl sulfate-polyacrylamide gel electrophoresis and transferred onto a polyvinylidene difluoride membrane (Millipore, Massachusetts, USA). Then, primary antibodies of BRD4 (Merck KGaA, Cat. No. PLA0227), ACSL3 (Merck KGaA, Cat. No. HPA071021), ACSL4 (Proteintech, Cat. No. 22401-1-AP), ACSL5 (Proteintech, Cat No. 15708-1-AP), ACSL6 (Abcam, Cat. No. ab229937), SRPK2 (Abcam, Cat. No. ab251113), p-SRPK2 (CST, Cat. No. 23708), mTOR (CST, Cat. No. 2983), p-mTOR (CST, Cat. No. 5536), p70 S6K (CST, Cat. No. 9202), p-p70 S6K (CST, Cat. No. 97596), and SRSF2 (Merck KGaA, Cat. No. HPA049905) were diluted to a working concentration and incubated overnight at 4 °C. Subsequently, the corresponding horseradish peroxidase (HRP)-conjugated secondary antibodies were incubated at 25 °C for 1 h, after which signals were detected by chemiluminescence using a Chemidoc system (Bio-Rad, Munich, Germany). Data were finally analysed using ImageJ software V1.8.0 (National Institutes of Health, Maryland, USA).

### Cell models with function-gain or function-loss of target genes

The coding sequences (CDS) of individual transcripts of ACSL3 and BRD4 were synthesized into the expression vector pcDNA3.1 according to demands for the research situation, while the short hairpin RNA (shRNA) targeting the CDS of BDR4, SRPK2 and SRSF2 were introduced into an interference vector hU6. Cell samples were collected 48 h after vector transfection, and the mRNA and protein levels of each target gene were detected by RT-qPCR and western blotting to measure the successful establishment of functional change cell models.

### Cell viability assay

SaoS2 or U2-OS cells with function-gain or function-loss of each target gene were plated into 96-well plates, then erastin was added to the cells in desired concentrations and left to stand for 24 h. Cell viability was then detected using an MTT assay kit following the manufacturer’s instructions (Merck, Cat. NO. 11465007001), after which absorbance was measured at 450 nm via a microplate reader (Bio-Rad Laboratories, Cat. NO. 1681135).

### Lipid ROS detection

The lipid ROS in living cells was detected by flow cytometry. Briefly, a 10 μmol/L boron-dipyrromethene C-11 probe (Millipore, Cat. No. MX5211) was added and incubated with cells in a 5% CO_2_ incubator at 37 °C for 1 h. Then, flow cytometry (Beckman, Cat. NO. DxFLEX) was used for the detection of intracellular lipid ROS. For the lipid ROS in tumor tissues, make the frozen section of tissue first, and then hybridize it with a boron-dipyrromethene C-11 probe on the section. Briefly, fresh tumor tissues were fixed with 4% paraformaldehyde for more than 24 h and then placed in 30% sucrose solution in a 4 °C refrigerator to dehydrate. Then the dehydrated tissues were taken out and O.C.T. Compound (Tissue-Tek, Sakura-Americas, USA) embedding agent was dropped around them. When O.C.T. turned white and hardened, sectioning was performed, followed by probe hybridization. Slivers were then scanned for each channel through whole slide imaging (3D-HISTECH, Cat. No. P250 FLASH) for lipid ROS analysis.

### MDA assay

Mouse tumor tissue samples were ground into tissue homogenates or osteosarcoma cell samples were lysed, followed by centrifugation (10,000 × *g*, 10 min) for collection of supernatant. MDA content in all samples was then tested and calculated according to the kit operating instructions (Beyotime, Cat. No. S0131S).

### Electron microscopy

Osteosarcoma cell samples were encapsulated and fixed in 1% agar. After dehydration, embedding and curing were conducted, followed by the preparation of ultrathin slides at 70 nm and staining with 2% uranium acetate and lead citrate. Finally, the images were taken using transmission electron microscopy (Hitachi, Cat. No. HT7800).

### In vivo xenograft tumor assay

Four-week-old male BALB/C nude mice (SJA laboratory animal CO. LTD, Hunan, China) were fed adaptively for seven days, after which they were injected with cell suspension (5 × 10^6^ per mouse) under the skin of the armpits to construct a mouse osteosarcoma tumor model. Then, tumor-forming mice were randomly assigned to receive tail vein administration at the period set by the protocol (*n* = 5). The injection dose comprised (+)-JQ1 (4 mg/mL, 50 μL/time) and erastin (1.75 mg/mL, 50 μL/time). The body weight of the mice was finally measured and recorded every five days during the observation. After the observation period, mice were euthanised using a one-time intraperitoneal injection of 7 mg/100 g excess pentobarbital solution, and tumor tissues were stripped for subsequent testing. The Experimental Animal Welfare ethical review consent of The Second Xiangya Hospital of Central South University (2022014) approved animal experiments.

### Determination of iron content (Prussian-blue staining)

The histological slides of tumor tissue samples were fixed with fixative for 30 s, rinsed with distilled water, and drained with filter paper. The smear was stained at 37 °C for 60 min (Abcam, Cat. No. ab150674) by dripping or immersing the working solution, then thoroughly rinsed with distilled water for 5 min. The water was drained with filter paper and counterstained with a nuclear solid red solution for 1–2 min. The smear was rinsed with distilled water and examined by microscopy after drying. Staining interpretation: nuclei appear red, cytoplasm pink, and iron bright blue.

### Identification of different transcripts of ACSL3

In brief, the total mRNA extracted from the cell sample was reversely transcribed into cDNA, and the amplification primers of ACSL3 covering the variable splicing region (forward: 5ʹ-GTTTGGCTTCAGTATTATACCCT-3ʹ and reverse: 5ʹ-TTCGAGTTCAGTTAGTTCCT-3ʹ) were designed. The differences in the molecular weight of ACSL3 fragments in the amplified products were examined by RT-qPCR and agarose gel electrophoresis.

### Measurement of mRNA stability

To measure mRNA stability, transcription was blocked by actinomycin D (5 µg/ml) treatment for 0, 2, and 4 h. Reverse transcription was performed using the same volume of RNA for all time points and the mRNA levels were measured by RT-qPCR.

### Immunofluorescence (IF)

The cell slides were fixed with 4% paraformaldehyde for 15 min in the culture plate and then permeated with 0.5% Triton X-100 at 25 °C for 15 min before use. The diluted ACSL3 primary antibody (Merck KGaA, Cat. No. HPA071021) was dropped and incubated at 4 °C overnight, the diluted fluorescence-labeled secondary antibody was dropped and incubated at 37 °C for 1 h, and DAPI was dropped and incubated at 25 °C for 5 min to stain the nucleus. All operations after the secondary antibody was dropped were performed in the dark. The residual liquid on the slides was washed with saline before each of the above procedures. For the final step, typical images were taken using a laser confocal instrument (SUNNY, Cat. No. CSIM100/110, Beijing, China). The IF data was quantified using Image J software (version 1.8.0, National Institutes of Health, Bethesda, MD, USA), and the average fluorescence intensity was used to indicate the average intensity of ACSL3-positive signals in the cytoplasm and nucleus.

### Co-immunoprecipitation (Co-IP) and mass spectrometry (MS) analysis of BRD4 interactome

Co-IP and MS assay were used to analyze the proteins binding with BRD4. First, the expression vectors pcDNA3.1-BRD4 were constructed and transfected into SaoS2 and U2-OS cells, and the IP grade antibody of BRD4 (Merck KGaA, Cat. No. PLA0227) was used for the immunoprecipitation process. Immunoprecipitated proteins were then analyzed by immunoblotting or further processed for the MS analysis.

For MS analysis, the immunoprecipitated proteins were eluted from the beads by incubating with V5 peptide (Sigma-Aldrich) overnight at 4 °C, then were precipitated with trichloroacetic acid (TCA, 20% w/v), rinsed three times with acetone, and dried at 25 °C. The pellets were re-suspended in 50 µL resuspension buffer (8 M urea, 50 mM ammonium bicarbonate, and 5 mM DTT) and subjected to reduction and alkylation reaction. Briefly, 15 mM iodoacetamide was added to each sample for 30 min in the dark at room temperature, followed by the addition of another 5 mM DTT to quench the reaction. Samples were diluted to a final concentration of 1 M urea, and then subsequently digested with LysC and trypsin at 25 °C overnight. For SRPK2 identification from the immunoprecipitated proteins, western blotting was performed with the anti-SRPK2 antibody (Abcam, Cat. No. ab251113).

### RNA immunoprecipitation (RIP)

RIP assay was performed by adopting the Magna RIP Quad RNA kit (Merck KGaA, Cat. No. 17–704) referring to the protocols to determine whether SRSF2 binds to the spliced intermediates of pre-mACSL3. In short, the cell samples were lysed and incubated with magnetic beads (Thermo Fisher) conjugated via anti-SRSF2 (Merck KGaA, Cat. No. HPA049905) or IgG (CST, Cat. No. 3900) overnight. The eluted RNA was further purified by phenol/chloroform extraction and precipitated with ammonium acetate and ethanol. Input and immunoprecipitated RNAs were treated with DNase I (Sigma-Aldrich) and reverse-transcribed (Takara), and the resulting cDNA was analyzed by RT-qPCR as described above. The amount of transcripts (%) bound to the antibody was calculated: 100 × 2^[Ct(Input) - Ct(IP)]. Primer of pre-mACSL3 used in RIP assay: forward: 5ʹ-CTCACAAAATAAATA-3ʹ and reverse: 5ʹ-TGCAACCTCCACCTCCT-3ʹ.

### Yeast two-hybrid system

Briefly, the prey vector pGADT7-BRD4 containing different BRD4 domains and a bait vector pGBKT7- SRPK2 were constructed and transferred into yeast to observe the BRD4 domain that binds to SRPK2. If the yeast could grow and turn blue on the SD/-Trp/-Leu/-His/-Ade plate and self-activation can be excluded, indicating an interaction between BRD4 and SRPK2. Meanwhile, the prey and bait identities of SRPK2 and BRD4 were changed to verify their binding relationships again.

### Targeted fatty acid detection

Sci-tech Innovation Co., LTD. (Shan Dong, China) provided targeted fatty acid detection and analysis in this study. In brief, this assay comprised three main steps: extraction of lipid samples from cells, saponification and methyl esterification, then the absolute determination of target fatty acid content in samples by gas chromatography. The data acquisition instrument system (Agilent, Cat. No.7890 A) was finally used to collect and analyse the GC data.

### Tissue samples acquisition from patients

Cancer tissues and nearby osteogenic tissues from patients with osteosarcoma were obtained during surgical removal (*n* = 18). The inclusion criteria were patients diagnosed with osteosarcoma who had not previously received any therapeutic interventions. All patients signed informed consent, and obtained the ethical approval of the scientific research project of the Medical Ethics Committee of the Second Xiangya Hospital of Central South University and the approval number is (2022) No. (Research 023). Then, the detection results of target genes from the same patient were paired for correlation analysis.

### Statistical analysis

Results are presented as mean ± SD unless mentioned otherwise. While two-tailed unpaired Student’s *t* test determined the statistical significance for two-group comparison, one-way ANOVA with Tukey or Dunnett was conducted for multiple-group comparison using GraphPad Prism 8 (GraphPad software). Bivariate correlation analysis and the Wilcoxon signed-rank test were performed to analyze the correlation between two different indicators. Significance in all figures was indicated as follows: *: *P* < 0.05, **: *P* < 0.01, ***: *P* < 0.001, *n.s*.: not significant.

## Results

### BRD4 inhibition protects osteosarcoma cells from erastin-induced ferroptosis

Previous work has shown that BRD4 inhibition leads to reduced susceptibility of tumor cells to erastin-induced ferroptosis [21]. Therefore, we first examined whether ferroptosis in osteosarcoma cells conforms to this regulatory mechanism. Here, we present a partial list of small molecule inhibitors and their biological targets that significantly affected cellular susceptibility to erastin- or RSL3- induced ferroptosis. It can be observed that when the concentration of these inhibitors was set at 10 μM, they exhibited differential roles in antagonizing or promoting ferroptosis (Fig. S1A–C). Among these inhibitors, we focused on (+)-JQ1 due to its pronounced antagonistic effect on erastin-induced ferroptosis, while it does not exert a significant effect on RSL3-induced ferroptosis. This suggests that (+)-JQ1 may selectively inhibit erastin’s effects on ferroptosis. Additionally, treatment with (+)-JQ1 alone at concentrations ranging from 0–10 μM in osteosarcoma cell lines SaoS2 and U2-OS does not result in a significant decrease in cell viability whereas treatment with erastin at concentrations of 1.5 μM and 2.5 μM resulted in a respective 50% decrease in cell viability. Importantly, it is worth noting that as the concentration of (+)-JQ1 gradually increases (within the range of 0–5 μM), its antagonistic effect on erastin-induced ferroptosis (abbreviated as erastin-ferrop, unless otherwise specified) also increases, and reaching a stable antagonistic effect on erastin-ferrop at concentrations between 5 and 10 μM (Fig. S1D).

**Fig. 1:**
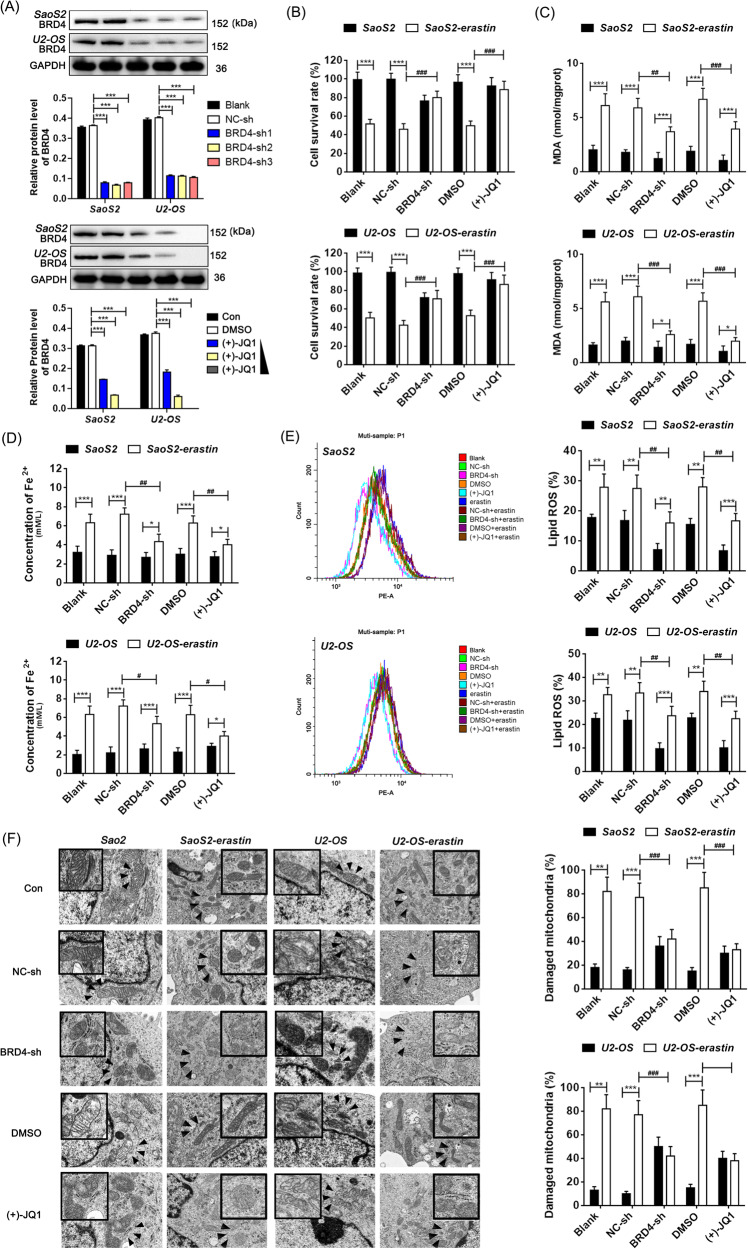
BRD4 inhibition protects osteosarcoma cells from erastin-ferrop in vitro. **A** Relative protein expression levels of BRD4 tested by western blotting in SaoS2 and U2-OS cells. **B** Cell survival rate analysis through the MTT assay. Intracellular MDA (**C**) and Fe^2+^ (**D**) content tested by analytical kits. **E** lipid ROS of each group determined by the boron-dipyrromethene C-11 probe and flow cytometry. **F** Electron microscopic images of mitochondria and the percentage of the damaged mitochondria. Tukey–Kramer test of one-way ANOVA, *: *P* < 0.05; **: *P* < 0.01; ***: *P* < 0.005.

Next, BRD4 function-loss models in osteosarcoma cell lines SaoS2 and U2-OS were conducted with shRNA or inhibitor (+)-JQ1 that targeting BRD4, and then cells were exposed to a culture medium containing erastin to induce ferroptosis (Fig. 1A and Fig. S2). Results showed that in addition to shRNA, (+)-JQ1 is also able to downregulate the expression of BRD4, which may be due to the inhibitor-induced endogenous degradation of BRD4. Moreover, inhibition of BRD4 either by shRNA or by (+)-JQ1 (10 μm) made cells more tolerant to erastin, which was manifested as an overall cell survival advantage, reduced MDA, lipid ROS and Fe^2+^ contents, and decreased mitochondrial damage rates (Fig. 1B–F), demonstrating the critical roles of BRD4 in erastin- ferrop of osteosarcoma cells.

**Fig. 2:**
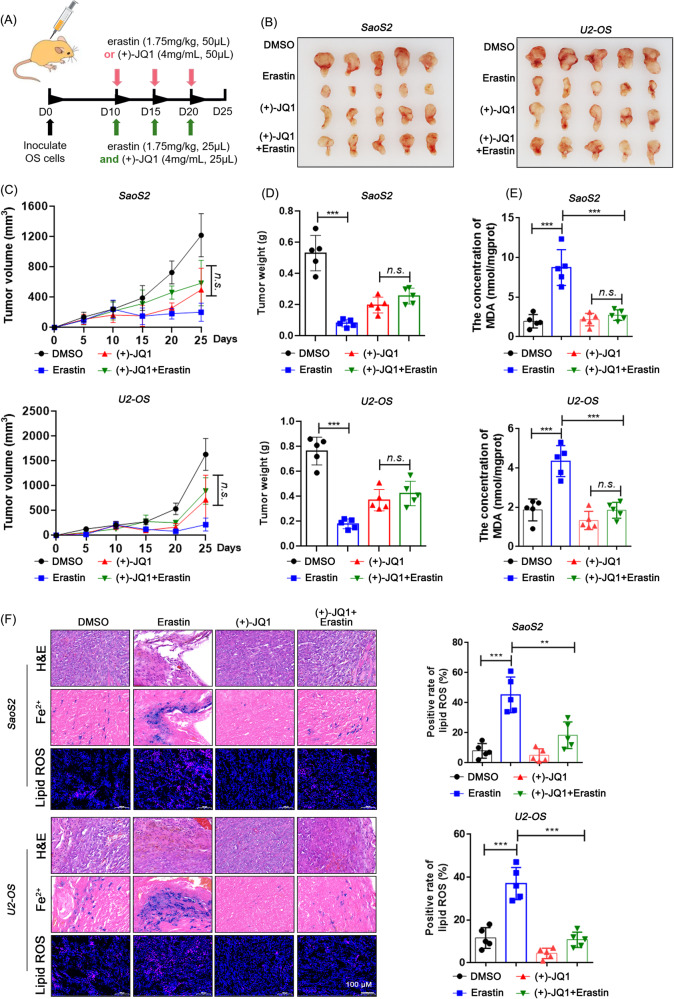
BRD4 inhibition protects osteosarcoma cells from erastin-ferrop in vivo. **A** Flowchart guide for the animal experiments. **B** Subcutaneous tumors from individual mice. Growth curve (**C**) and weights (**D**) of subcutaneous tumors. **E** MDA content in tumors. **F** Prussian blue staining of ferric ions and probe hybridization of lipid ROS in tumor tissue slides. Dunnett’s test of one-way ANOVA, *: *P* < 0.05; **: *P* < 0.01; ***: *P* < 0.005; n.s.: no significance.

Subcutaneous tumor models of osteosarcoma cells in nude mice were then constructed to evaluate the effects of BRD4 inhibition on erastin-ferrop in vivo (Fig. 2A). It was found that both (+)-JQ1 and erastin were effective in halting the growth of tumor mass, but their effects were weakened when used in combination (Fig. 2B–D). In addition, erastin in the (+)-JQ1 processing group could not cause the contents of Fe^2+^, lipid ROS and MDA to rise as those in the non- (+)-JQ1 processing group (Fig. 2E, F), suggesting that (+)-JQ1 may limit erastin’s antitumor effects in vivo.

Collectively, inhibiting BRD4 helps protect osteosarcoma cells from erastin-ferrop at both in vivo and in vitro levels.

### BRD4 is involved in post-transcriptional regulation of ACSL3 to ensure the expression abundance of ACSL3

Mammals express multiple isoforms of acyl-CoA synthetase (ACSL1 and ACSL3–6) in various tissues [22]. To identify ACSLs that regulated by BRD4, changes in mRNA and protein expression levels were assessed in osteosarcoma cells before and after treatment with (+)-JQ1. Results showed that, except for ACSL1, mRNA and protein levels of ACSL3-6 all exhibited varying degrees of decrease, suggesting that the inhibition of BRD4 may have a global impact on the ACSL family (Fig. S3). The most important motivation behind our interest in the potential relationship between ACSL3 and BRD4 stems from previous reports suggesting that inhibition of either ACSL3 [20] or BRD4 [21] is associated with cellular ATP depletion, which may have an impact on erastin- ferrop.

**Fig. 3:**
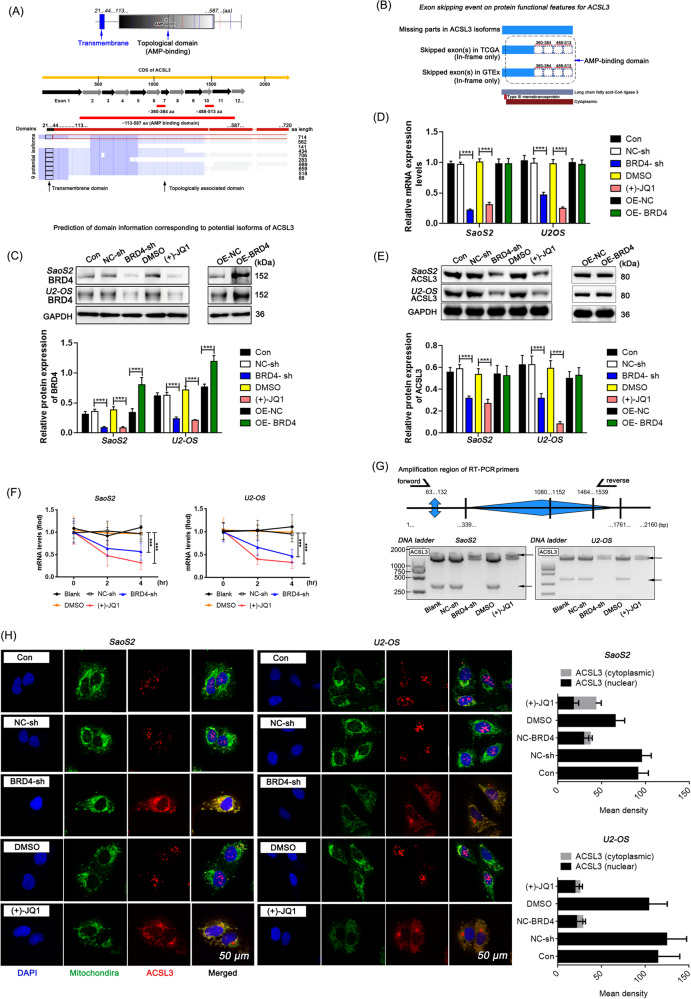
The dual effects of BRD4 on ACSL3 expression and subcellular location. **A** Functional domains of the complete ACSL3 protein with its corresponding aa length and the alignment result of the aa sequences for nine potential isoforms of ACSL3 collected from the *UniProt* database. **B** The genomic structures of ACSL3 exon skipping events of *TCGA* and *GTEx* across reference gene model from the *ExonskipDB* database. **C**, **E** Relative protein levels detected by western blotting. **D** Relative mRNA levels detected by RT-qPCR. **F** RNA stability assessed by RNA digestibility tests. **G** Detection of splicing variants of ACSL3 via RT-qPCR and agarose gel electrophoresis. Dunnett’s test of one-way ANOVA, *: *P* < 0.05; **: *P* < 0.01; ***: *P* < 0.005. **H** Typical images of ACSL3 and mitochondrial localization captured by laser confocal microscopy.

The amino acid sequence alignment results of the nine potential isoforms of ACSL3 collected in the *Uniprot* database up to now and the genomic structures of exon skipping events of *TCGA* and *GTEx* across reference gene model from the *ExonskipDB* database were presented (Fig. 3A, B). Given that most pre-mRNA splicing events occur during co-transcription [23], we first examined the effect of BRD4 on ASCL3 expression. The result supports that inhibition of BRD4 leads to a significant decrease in the total mRNA and protein levels of ACSL3. However, the up-regulation of BRD4 did not have the expected up-regulation effect on the expression of ACSL3 mRNA and protein (Fig. 3C–E and Fig. S4). Moreover, the stability of ACSL3 mRNA was under BRD4’s marked influence and reduced significantly after BRD4 inhibition (Fig. 3F). These findings suggest that BRD4 may be a necessary but not sufficient condition for ACSL3 transcription activation, and also crucial for maintaining the stability of ACSL3 transcription products to ensure optimal expression abundance.

We subsequently examined the impact of BRD4 inhibition on the expression abundance of different transcript variants of ACSL3, and results showed that the abundance of transcripts that could be amplified by amplification primers at either end of the AMP-binding domain decreased significantly in cell samples with BRD4 inhibition, as did the abundance of two common exon skipping transcripts (Exon-skip-33420, Exon-skip-33428) (Fig. 3G), implying a global impact of BRD4 inhibition on post-transcriptional control of ACSL3. Another noteworthy phenomenon is that BRD4 also affects the subcellular localization of ACSL3. Specifically, ACSL3 is generally scattered in the nucleus or cytoplasm in osteosarcoma cells, but when BRD4 is interfered, ACSL3 is more dispersed in the cytoplasm with a decrease in overall protein levels (Fig. 3H). These data demonstrate the significance of BRD4 in maintaining the abundance of ACSL3 expression. Inhibiting BRD4 reduces ACSL3 expression and ultimately leads to alterations in its spatial localization, which may directly impact the biological function of ACSL3.

### Elevating the full-length transcript of ACSL3 increased the arachidonic acid content and erastin-ferrop susceptibility in osteosarcoma cells

**Fig. 4:**
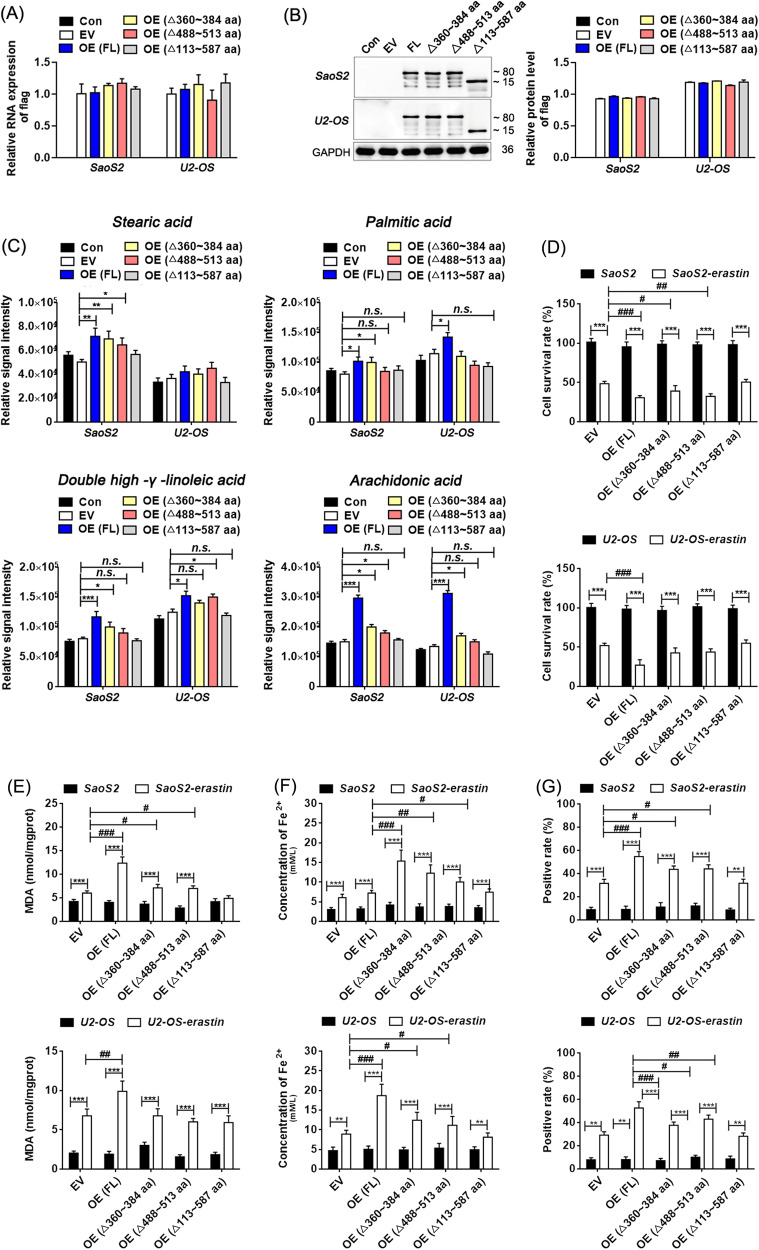
Elevating the full-length transcript of ACSL3 increased the arachidonic acid content and erastin-ferrop sensitivity. **A** Relative mRNA levels detected by RT-qPCR. “△“ means absence. **B** Relative protein levels detected by western blotting. **C** Abundance analysis of intracellular stearic acid, palmitic acid, double high -γ -linoleic acid and arachidonic acid by targeting fatty acid metabolomics. **D** Cell survival rate. Intracellular content of MDA (**E**), Fe^2+^ (**F**), and lipid ROS (**G**). Dunnett’s test of one-way ANOVA, *: *P* < 0.05; **: *P* < 0.01; ***: *P* < 0.005; *n.s*.: no significant.

The role of the AMP-binding domain of ACSL3 on erastin-ferrop was focused on this study, not only because it is involved in energy metabolism signaling, but also because it constitutes different ACSL3 splicing variants and protein isoforms. The expression vectors of ACSL3 with the full-length transcript, the AMP-binding domain-loss transcript (△113 ~ 587 aa), and the two common ACSL3 exon skipping transcripts with a flag tag were constructed and transfected into osteosarcoma cells respectively. Then the contents of unsaturated fatty acids were detected, including stearic acid, palmitic acid, double high -γ -linoleic acid, and arachidonic acid that might be substrates of ACSL3 and were proposed as participants in ferroptosis affected by AMP-activated protein kinase (AMPK) signaling [24]. Results showed that the mRNA and protein levels of the label molecule flag were significantly increased in the recipient cells (Fig. 4A, B, Fig. S5), proving the success of the fusion expression of ACSL3 and GFP. Over-expression of ACSL3 had the greatest positive effect on arachidonic acid, especially when the full-length transcript of ACSL3 was imported externally. While other transcripts, especially those with AMP-binding domain deficiency, had little effect on it (Fig. 4C). Moreover, cells in the full-length transcript over-expression group were more sensitive to erastin, as showed by lower cell viability and more MDA, lipid ROS and Fe^2+^ contents compared with other transcripts over-expressed groups (Fig. 4D-G). In particular, over-expression of ACSL3 with AMP-binding domain deletion had almost no effect on arachidonic acid and erastin-ferrop, an indication of that maintaining ACSL3 with full biological function at a certain level is beneficial for inducing erastin-ferrop in osteosarcoma cells.

**Fig. 5:**
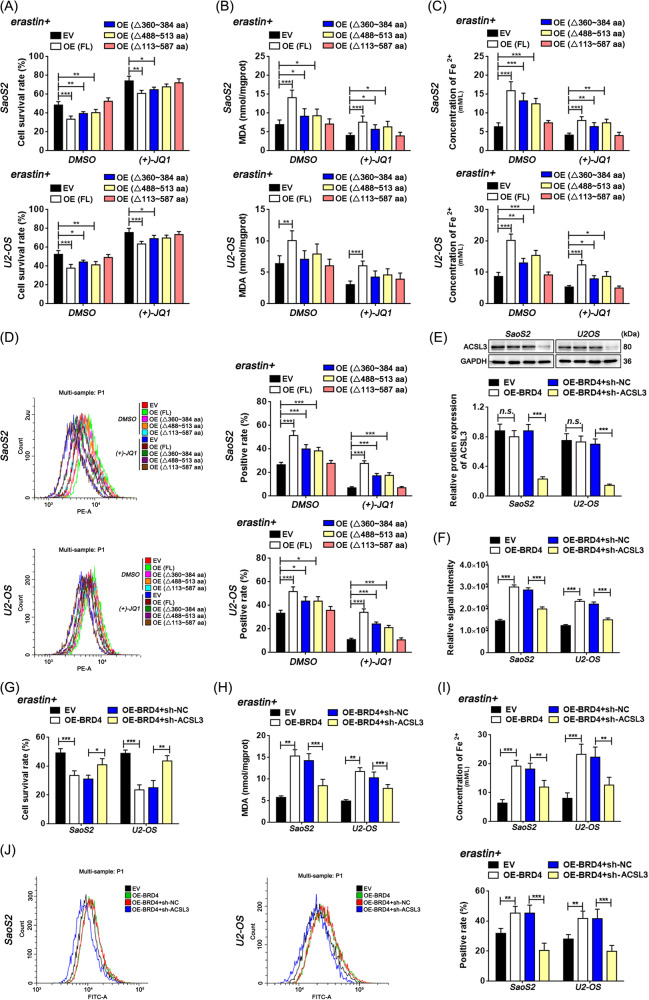
The effects of BRD4 on erastin-ferrop are partly working through the ACSL3 pathway. **A**, **G** Cell survival rate. **B**, **H** Intracellular content of MDA. **C**, **I** Intracellular content of Fe^2+^. **E** Relative protein levels detected by western blotting. **F** Abundance analysis of intracellular arachidonic acid. **D**, **J** Positive rate of intracellular lipid ROS. Dunnett’s test of one-way ANOVA, *: *P* < 0.05; **: *P* < 0.01; ***: *P* < 0.005; *n.s*.: no significant.

Then we investigated whether BRD4 exerted its influence on erastin-ferrop through ACSL3. Results indicated that the decreased susceptibility to erastin-ferrop, caused by the inhibition of BRD4, could be partially restored with upregulated full-length transcripts of ACSL3 (Fig. 5A–D and Fig. S6). Conversely, knocking down ACSL3 in osteosarcoma cells overexpressing BRD4 revealed that the accumulation of arachidonic acid and erastin-ferrop induced by BRD4 can be further attenuated by ACSL3 inhibition (Fig. 5E–J). This suggests that the impact of BRD4 on erastin-ferrop is partially mediated through the ACSL3-dependent pathway.

To conclude, the AMP-binding domain is essential for ACSL3 participating in arachidonic acid synthesis and erastin-ferrop that affected by BRD4.

### BRD4 recruits SRPK2 to catalyse the splicing efficiency of pre-mACSL3

**Fig. 6:**
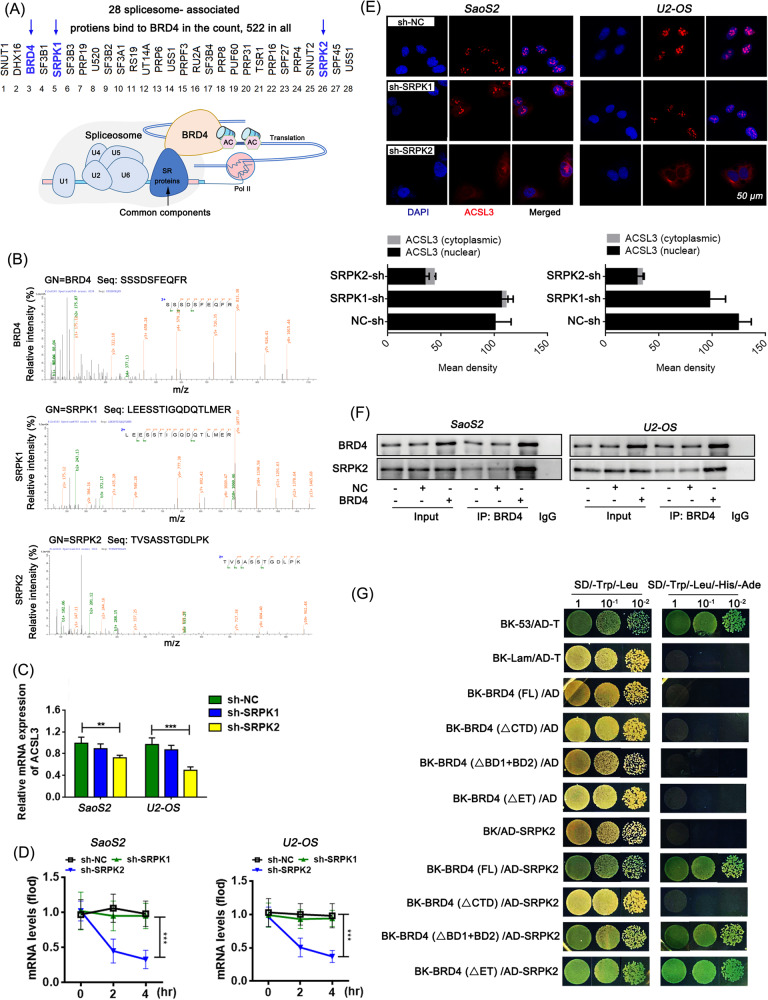
SRPK2 is recruited by BRD4 and binds to its CTD domain. **A** 28 splicesome-associated proteins that bind to BRD4 via Co-IP and MS assay. **B** Unique sequences of SRPK1 and SRPK2 that bound to BRD4 protein analyzed by MS. **C** Relative mRNA levels detected by RT-qPCR. Dunnett’s test of one-way ANOVA, *: *P* < 0.05; **: *P* < 0.01; ***: *P* < 0.005. **D** RNA stability assessed by RNA digestibility tests. **E** IF staining results of ACSL3 in cells, typical images taken with laser confocal microscopy. **F** Endogenous binding relationship of BRD4 and SRPK2 identified by Co-IP method. **G** Yeast hybrid system to verify the binding domain of BRD4 (BD1, BD2, ET, and CTD domains) to SRPK2.

To investigate the possible mechanisms of BRD4 regulating the post-transcription of ACSL3, we screened the splicesome-associated proteins that BRD4 may work together via Co-IP and MS assay, and a total of 28 splicesome-associated proteins were found (Fig. 6A). We noticed that the SRSF protein kinase (SRPK) 1 and 2, the two highly specific protein kinases for the Serine/arginine (SR)-rich family of splicing factors [25], were both present in the BRD4-immunoprecipitate products with unique bounding peptides (Fig. 6B). By knocking down SRPK1 or SRPK2 respectively, we found that the ACSL3 expression and stability were significantly affected by SRPK2, but not by SRPK1 (Fig. 6C–E). Further, the endogenous binding relationship between SRPK2 and BRD4 in osteosarcoma cells was verified and the main binding site was analyzed in the CTD domain of BRD4 (Fig. 6F, G, Fig. S7).

**Fig. 7:**
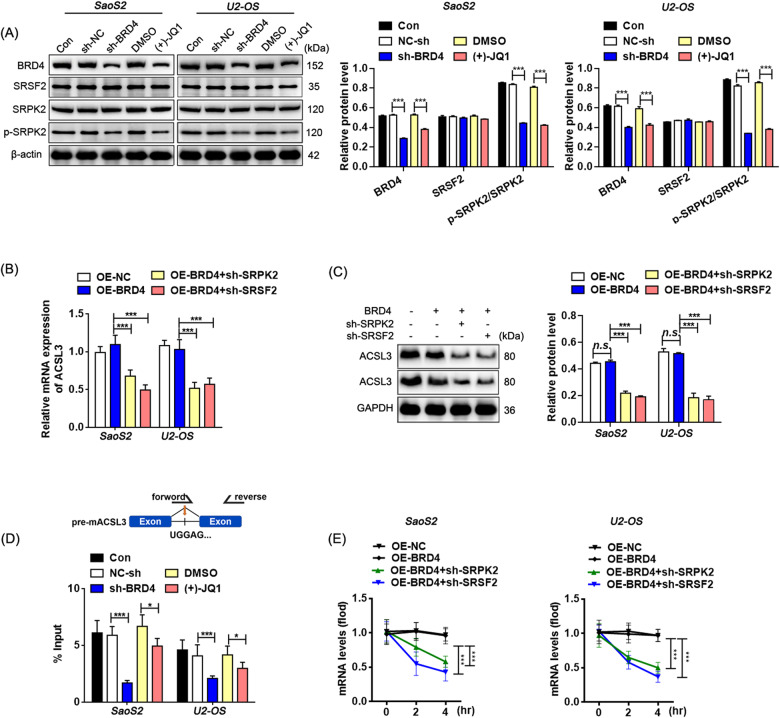
BRD4 affects splicing efficiency of pre-mACSL3 through SRPK2. **A**, **D** Relative protein levels detected by western blotting. **B** The enrichment of ACSL3 mRNA in the immunoprecipitate product of anti-SRSF2 antibody via the RIP/ RT-qPCR assay. **C** Relative mRNA levels detected by RT-qPCR. **E** RNA stability assessed by RNA digestibility tests. Dunnett’s test of one-way ANOVA, *: *P* < 0.05; **: *P* < 0.01; ***: *P* < 0.005.

The effects of the BRD4-SRPK2 complex on ACSL3 splicing and expression were then demonstrated, and it was found that inhibition of BRD4 had no significant effect on protein expression of SRPK2 and SRSF2, but brought down phosphorylated SRPK2 notably (Figs. 7A, S8). Given that SRSF2 is a substrate of SRPK2 and is involved in pre-mRNA splicing [26], we did detect the intermediate mRNA fragments of pre-mACSL3 splicing from the mRNA products precipitated by the anti-SRSF2 antibody, and the content of which reduced significantly in cells with BRD4 inhibition (Fig. 7B), supporting an SRSF2-mediated pre-mACSL3 splicing controlled by BRD4. In addition, the down-regulation of SRPK2 or SRSF2 in BRD4 over-expressed cells led to a significant reduction of ACSL3 mRNA and protein expression levels and a decline in ACSL3 mRNA stability (Fig. 7C–E, Fig. S8), interpreting as that BRD4 influences ACSL3 splicing and expression in the form of SRPK2 and SRSF2 dependencies.

**Fig. 8:**
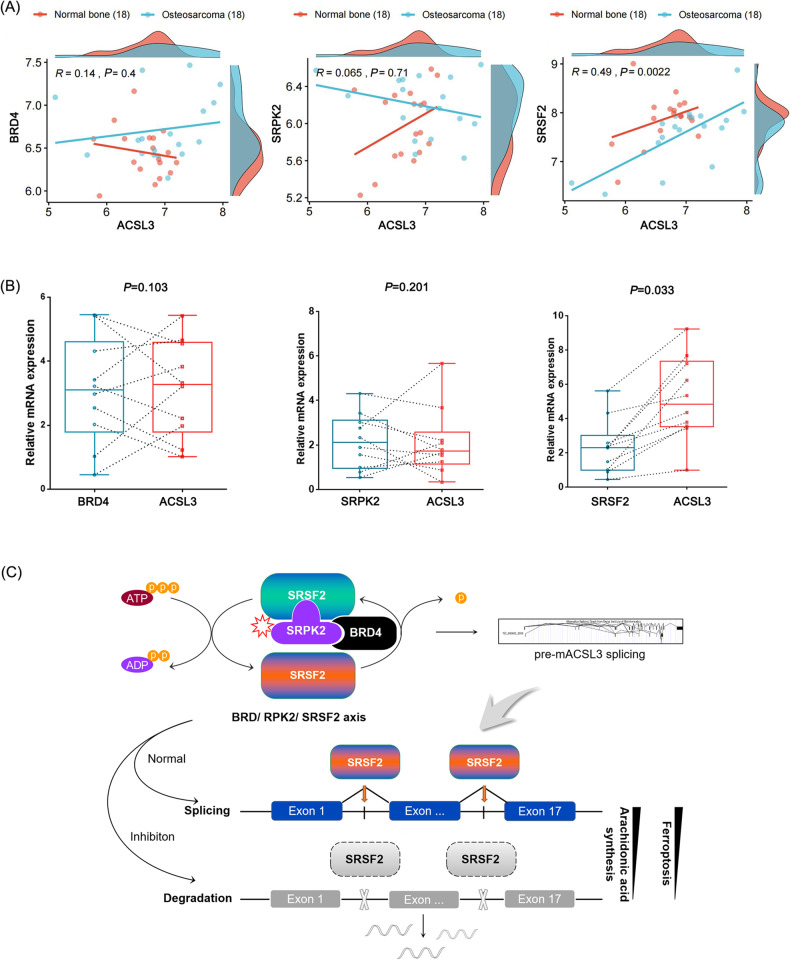
Correlation scatter plot between BRD4, SRPK2, SRSF2 and ACSL3 in osteosarcoma tissue. **A** Data from the *GEO* database (*n* = 18, Bivariate correlation analysis, *R* ≤ 0.4: low correlation; *R* å 0.4: middle correlation; *P* < 0.05: significant). **B** Intra- patient variation in diversity index (*n* = 10, Wilcoxon signed-rank test). **C** Schematic diagram of BRD4/SRPK2/SRSF2 axis in pre-mACSL3 splicing and expression.

Finally, 18 paired osteosarcoma tissues and normal osteogenic tissues were selected from the *GEO* database (cancerous tissue matched 1:1 with adjacent osteogenic tissue, GSE99671) to analyze the expression correlation between BRD4, SRPK2, SRSF2 and ACSL3. It shows that the expression of ACSL3 was not correlated with the expression of BRD4 and SRPK2, but positively correlated with SRSF2 (Fig. 8A), which is consistent with our findings in 10 cancerous tissues surgically removed from patients with osteosarcoma (Fig. 8B). Therefore, we can reach the following conclusion that although BRD4 or SRPK2 does not activate ACSL3 expression directly, they are essential to ensure the SRSF2-mediated splicing efficiency of pre-mACSL3 and then affect the final expression abundance of ACSL3 (Fig. 8C).

## Discussion

Cancer cells regulate the synthesis of macromolecules to meet the need for continuous proliferation [27]. By enhancing the uptake of exogenous lipids and mediating lipid de novo synthesis, cancer cells can be helped to expand cellular and organellar membranes. In this process, fatty acids are at the crossroads of lipid anabolic and catabolic pathways, as they can participate in the synthesis of phospholipids and triacylglycerol, and can also undergo beta-oxidation to be broken down [28]. Key to the decisions are long-chain acyl- CoA synthetases (ACSLs), enzymes that catalyze the activation of long-chain fatty acids of 12–22 carbons [29].

ACSLs play important roles in lipid metabolism, with different subtypes exhibiting distinct tissue distribution and substrate preferences, thus regulating the lipid composition within cells. Among 5 subtypes of ACSLs, ACSL3 and ACSL4 have been found to be associated with ferroptosis [30]. However, limited information is available regarding the specific roles of ACSL3 and ACSL4. According to existing research, ACSL3 and ACSL4 play differential roles in ferroptosis. Specifically, ACSL3 is indispensable in exogenous monounsaturated fatty acids (MUFAs)- induced ferroptosis resistance [31], while ACSL4 is indispensable for the biosynthesis of PUFAs and the response to ferroptosis [8]. Despite the proposal of an effective method to promote ferroptosis tolerance through increased intake of exogenous MUFAs depending on ACSL3, other studies have suggested a link between ACSL3 deficiency and ATP consumption in cancer cells, which may hinder the synthesis of PUFAs and be disadvantageous to ferroptosis, as the energy system is as vital as the enzymatic system in determining the PUFAs synthesis and ferroptosis.

The AMP-binding domain of ACSL3 is unconserved or even absent in different isoforms according to the known variant aa sequences, which means they may respond differently to AMPK signaling. In this study, we found that over-expression of the full-length transcript of ACSL3 most significantly increased intracellular arachidonic acid content than overexpression of other isoforms. One possible explanation for this is the change in substrate preference of the different variants. Besides, by upregulating ACSL3, cells become more sensitive to erastin-ferrop, and replenishing ACSL3 expression in cells partially restored the decreased susceptibility to erastin-ferrop caused by BRD4 inhibition. Taken together, our study findings indicate that the AMP binding domain of ACSL3 determines its impact on the biosynthesis of intracellular arachidonic acid (a long-chain polyunsaturated fatty acid that contributes to ferroptosis [32, 33]), thereby influencing erastin-ferrop. However, as with other proteins on the hydrolipid interfacial membrane, accurately assessing the biological activity of ACSL3 remains a challenge in the need for more advanced and precise technological approaches for support.

A lung cancer study showed that KAS mutations drive ACSL3 expression, thereby promoting fatty acid absorption, retention, accumulation, and beta-oxidation in cancer cells, and identified ACSL3 as a potential metabolic therapeutic target [20]. Besides, because of the N-terminal transmembrane hydrophobic domain of ACSL3 can anchor it to the cytoplasmic membrane, scholars have linked ACSL3 with unfolded protein response and found that ACSL3 supported endoplasmic reticulum (ER) function to provide advantages for cells to survive under ER stress [34, 35]. Therefore, it has been proposed that inhibition of ACSLs may be particularly suitable for killing cancer cells dependent on fatty acid resynthesis, because it can overcome the compensatory activation of another pathway brought about by single inhibition of exogenous lipid uptake or fatty acid neovascularization, thereby inducing a more effective antitumor response [29]. However, it is a pity that no targeted inhibitors of ACSL3 are available yet. An alternative is to inhibit ACSL3 with mammalian target of rapamycin (mTOR) inhibitors according to the presence of sterol regulatory element binding protein 1 (SREBP1) element in the promoter region of ACSL3 [20]. In the present study, we add the significance of targeting BRD4 for ACSL3 inhibition. Mechanically, the SRPK2-SRSF2 executive platform for pre-mACSL3 splicing could not operate effectively when BRD4 is restricted, because SRPK2 is recruited by BRD4 to catalyze the splicing activity of SRSF2.

BRD4 is the most well-studied epigenetic “reader” in the BET family [36]. Currently, there is debate about the effect of BRD4 on ferroptosis. Some studies have suggested that BRD4 suppresses ferroptosis by transcriptionally promoting the expression of glutathione peroxidase 4 (GPX4), solute carrier family 7 member 11 (SLC7A11) and solute carrier family 3 member 2 (SLC3A2) and inhibiting the autophagic degradation of ferritin [37]. But according to our previous study, inhibition of BRD4 reduces erastin-ferrop susceptibility of tumor cells via blocking the expression of nuclear transcription mitochondrial lipid metabolism genes including hydroxyacyl-CoA dehydrogenase (HADH), acyl-CoA synthetase long-chain family member 1 (ACSL1) and acetyl-CoA acyltransferase 2 (ACAA2) [21]. The contradictory results suggest the multifaceted role of BRD4 in ferroptosis. In this study, we report a splicing mechanism regulated by BRD4, which mediates the splicing efficiency of ACSL3 and promotes the synthesis of arachidonic acid, thereby ensuring cellular susceptibility to erastin-ferrop. Therefore, we propose that the impact of BRD4 on ferroptosis is multifaceted and should be separately discussed in the context of different disease types and ferroptosis inducers.

Cells use a variety of post-transcriptional mechanisms to fine-tune mRNAs and generate proteomic diversity, such as splicing, capping, polyadenylation, methylation, nuclear export, and stability regulation [38]. These processes are regulated to some extent by various RNA-binding proteins, such as SR-rich proteins and hnRNPs [39]. SR-rich proteins are encoded by the Serine/arginine-rich splicing factor (SRSF) genes and recruit proteins such as small nuclear ribonucleoproteins (snRNPs) to catalyze mRNA processing [40]. SRSF2, a splicing factor that widely expressed in a variety of mammalian cell types, acts as an important sensor and effector during multiple disease progression [41]. Studies have shown that the activity of SRSF2 can be regulated by phosphorylation of the RS domain by SR protein kinase 2 (SRPK2) [42], and then binds normally to the exon splicing enhancers to regulate exon splicing of pre-mRNAs [43]. In this study, we found that pre-mACSL3 splicing efficiency is BRD4-SRPK2-SRSF2 axis dependent. To be specific, SRSF2 is directly responsible for pre-mACSL3 splicing, while BRD4 recruits SRPK2 to catalyze the splicing efficiency of SRSF2, which is necessary for intracellular arachidonic acid synthesis. These data fill some of the gaps in understanding the post-transcriptional regulatory mechanisms of ACSL3.

It is worth mentioning that a molecule that consistently emerges in numerous studies when investigating lipid metabolism from the perspective of pre-mRNA splicing is the mechanistic target of rapamycin complex 1 (mTORC1). Research has shown that ACSL3 transcription is activated by mTORC1 signaling [20], while BRD4 becomes more stable after mTORC1 signaling activation [44]. Moreover, SRPK2 acts as an effector in response to post-transcriptional splicing of lipid metabolism genes controlled by mTORC1 [45]. Therefore, it can be hypothesized that upon the requirement for lipid synthesis, mTORC1 signaling is initially activated, promoting the transcription of ACSL3. Subsequently, pre-mACSL3 splicing mediated by BRD4 and SRPK2 ensures the expression abundance of ACSL3 in cancer cells. However, as a hub of cellular nutrition and energy metabolism, mTORC1 signaling determines the ferroptotic response in a context-dependent manner [46]. Some scholars posit that inhibiting mTOR helps to stimulate ferroptosis in cells, as mTOR is responsible for the synthesis of key protein molecules in the antioxidant system, including GPX4, among others [47, 48]. Conversely, other scholars have discovered that inhibiting the mTORC1 by activating the AMPK signaling pathway can protect cells from ferroptosis by reducing lipid synthesis [24]. In this study, we created a glucose-starvation environment to inhibit mTORC1 activity in cells to view the impact on the BRD4-SRPK2-SRSF2 signal axis and found a significant decline in the expression of BRD4, ACSL3 and the phosphorylated SRPK2. Meanwhile, the binding of SRSF2 on pre-mACSL3 splicing intermediates decreased significantly when mTORC1 was inhibited by its inhibitor, rapamycin, implying a mTORC1-dependent BRD4-SRPK2-SRSF2 axis mediating pre-mACSL3 splicing (Fig. S9). Our findings are consistent with the observations of Hyemin Lee et al. [24], suggesting that inactivation of mTOR signals due to energy stress can lead to insensitivity to erastin-ferrop.

In general, cancer cells are frequently confronted with energy stress due to their rapid growth and limited oxygen supply, which inhibits the activity of mTOR signaling and potentially weakens the regulatory role of the BRD4-SRPK2-SRSF2 axis on ACSL3 expression. This restricts the growth and metabolism of cancer cells, while also forcing them to undergo metabolic reprogramming to adapt to the stressful environment, resulting in alterations in their susceptibility to erastin-ferrop. Therefore, identifying key molecules linking metabolism and ferroptosis may serve as highly promising biological markers or targets in the development of anticancer strategies.

## Conclusion

In this study, we found that BRD4-SRPK2-SRSF2 constructed a processing platform of pre-mACSL3 splicing to ensure the expression abundance of ACSL3 in osteosarcoma cells, thus playing important roles in arachidonic acid synthesis based on its AMP-binding domain and subsequently impacting susceptibility to erastin-ferrop.

### Supplementary information

Supplemental Materials

Response to aj-checklist

## Data Availability

All data generated or analyzed during this study are included in this article. The datasets used and/or analyzed during the current study are available from the corresponding author upon reasonable request.
