# Supplementary material for: The BRD4-SRPK2-SRSF2 signal modulates the splicing efficiency of ACSL3 pre-mRNA and influences erastin-induced ferroptosis in osteosarcoma cells
Source: Cell Death Dis. 2023 Nov 23;14(11):760. doi: 10.1038/s41419-023-06273-2 (PMC10665344; doi:10.1038/s41419-023-06273-2)
Supplement: Supplementary file 1 — Supplemental Materials [file 41419_2023_6273_MOESM1_ESM.pdf]

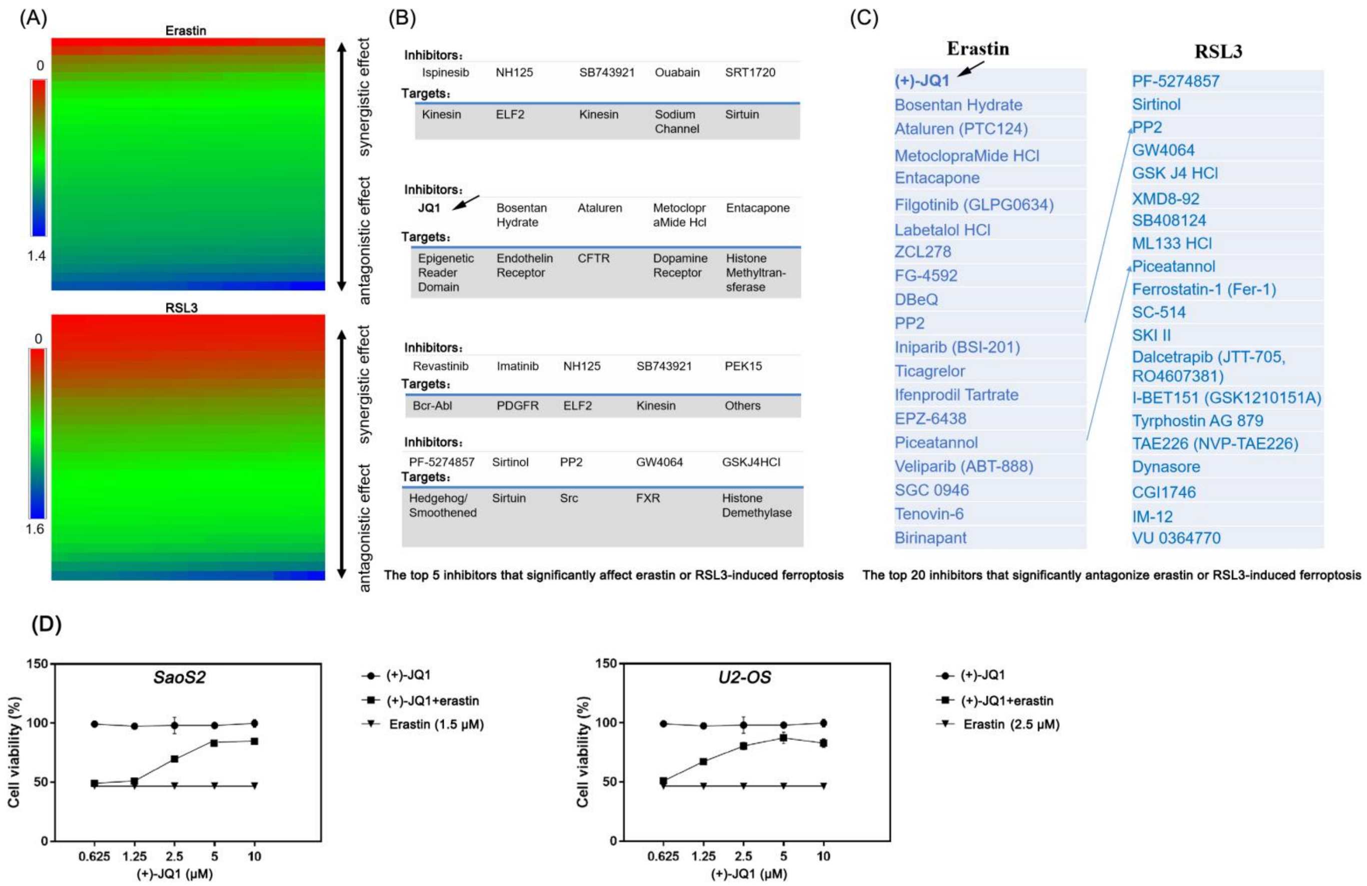

Fig. S1 Supplemental information on small molecule inhibitors with significant effects on erastin and RSL3-induced ferroptosis. (A) Heat maps of the OD values showing the MTT screening results of cells treated with 464 inhibitors and ferroptosis inducers: erastin or RSL3. OD = 0.5 is the dividing line, the reference OD value of cells treated with erastin and fer-1 (a ferroptosis inhibitor). Among these inhibitors, while 0.1 < OD < 0.9 (green) indicated no significant effect of inhibitors on ferroptosis, OD ≤ 0.1 (red) and OD ≥ 0.9 (blue) were significantly promoted or antagonised ferroptosis. (B) The top 5 inhibitors that significantly promote or antagonize erastin- or RSL3-induced ferroptosis and their biological targets. (C) The top 20 inhibitors that significantly antagonize erastin and RSL3-induced ferroptosis. Connect the same inhibitors with blue arrows: PP2 (target: Src) and Piceatannol (target: Syk). The black arrow indicates the most effective inhibitor against erastin-induced ferroptosis: JQ1. (D) Effects of JQ1 (0-10 μM) and erastin alone and in combination on cell viability measured by MTT.

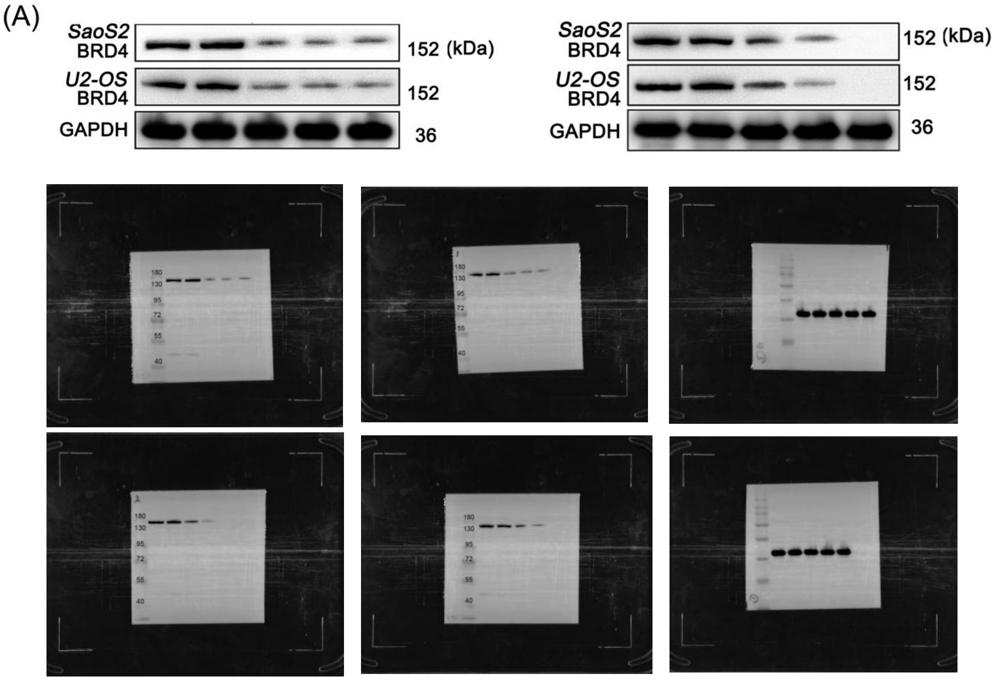

Fig. S2 Supplementary information of western blots in Fig. 1A.

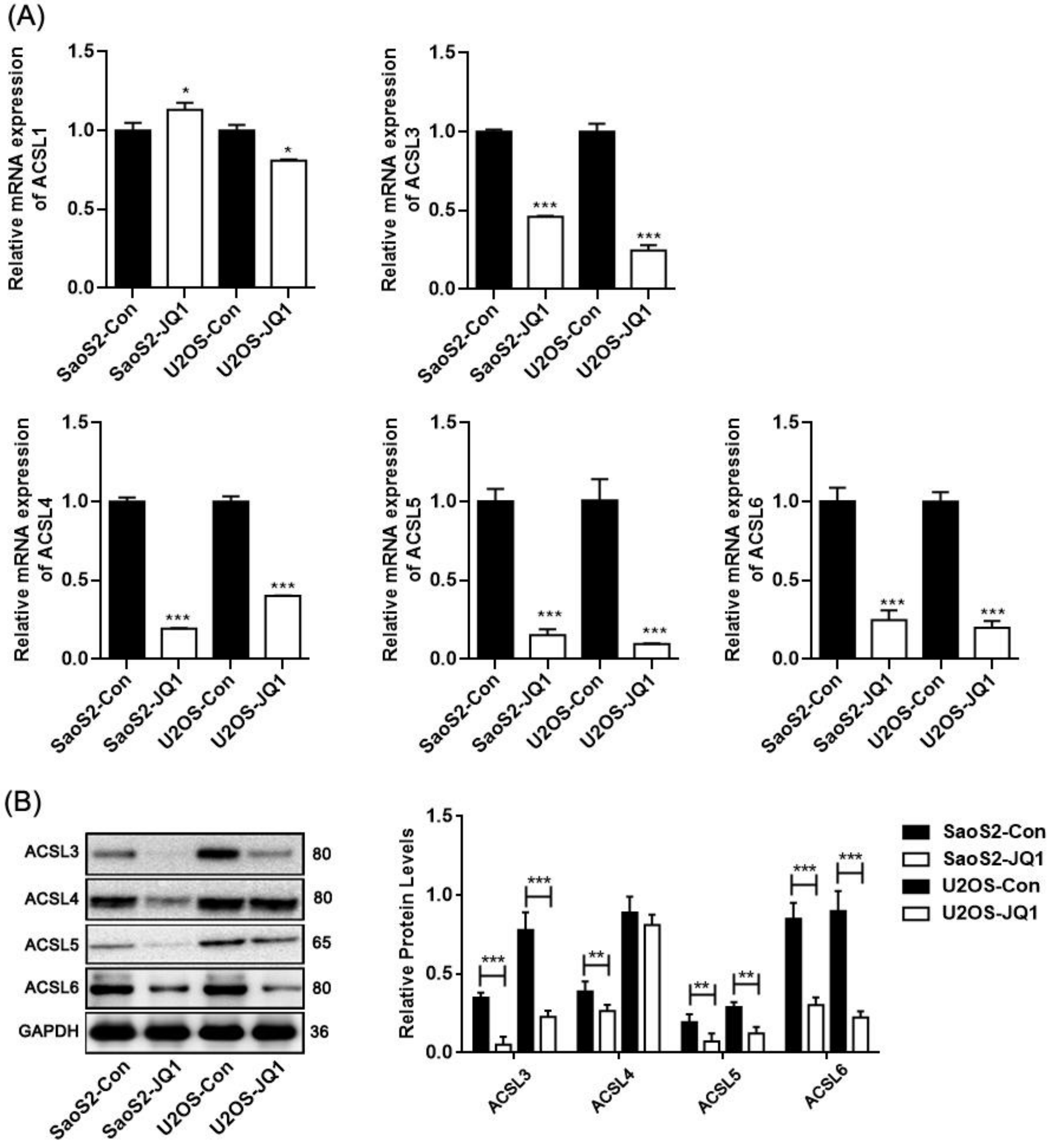

Fig S3. Supplementary information of the effects of JQ1 on the expression level of ACSLs. (A) Relative mRNA expression levels detected by RT-qPCR. (B) Relative protein expression levels detected by western blotting. Student's *t* test, *: $P < 0.05$; **: $P < 0.01$; ***: $P < 0.005$.

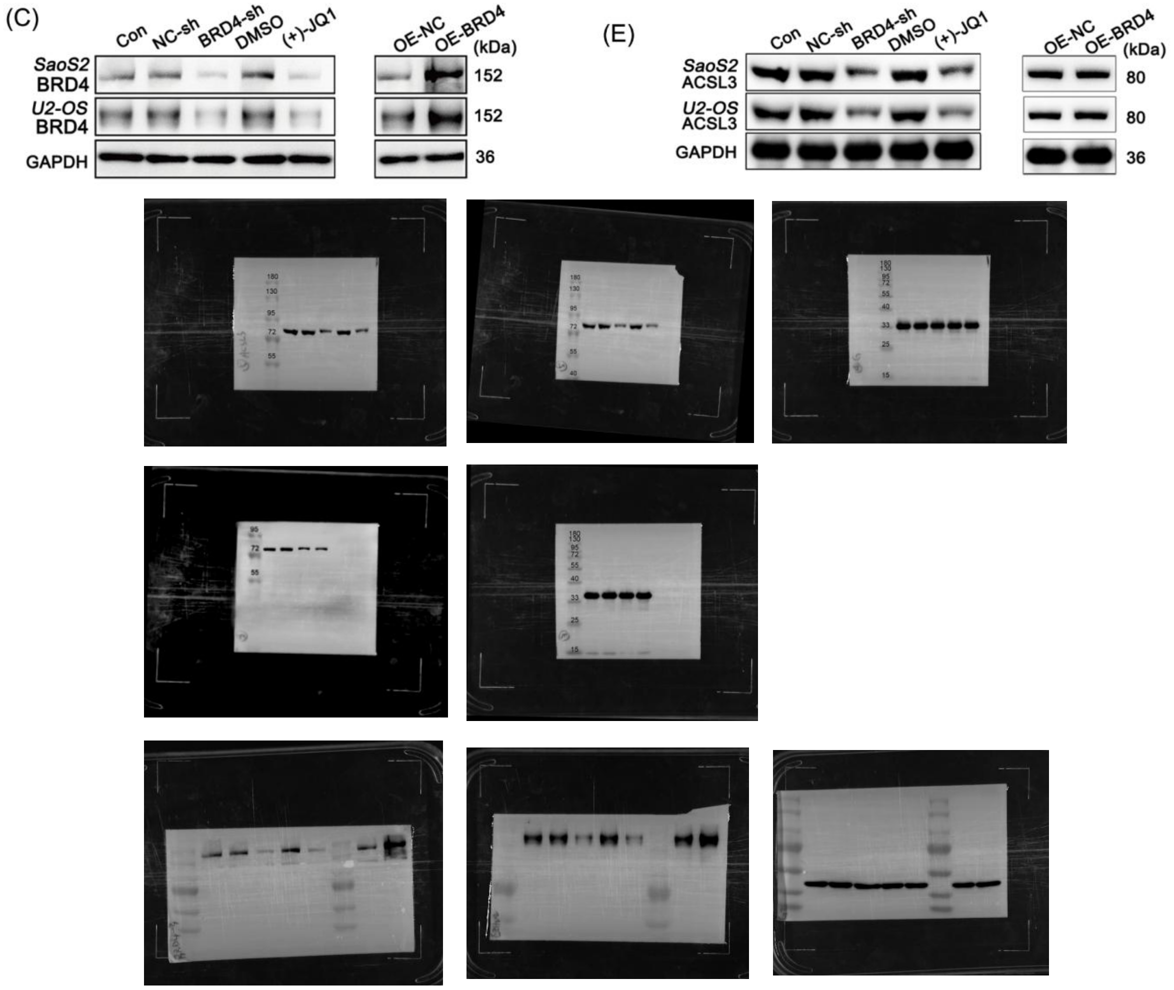

Fig. S4 Supplementary information of western blots in Fig. 3C and 3E.

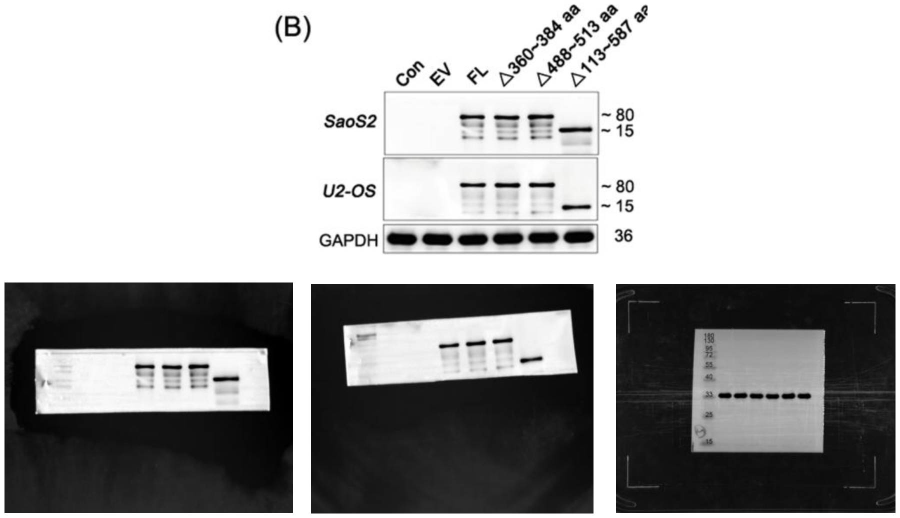

Fig. S5 Supplementary information of western blots in Fig. 4B.

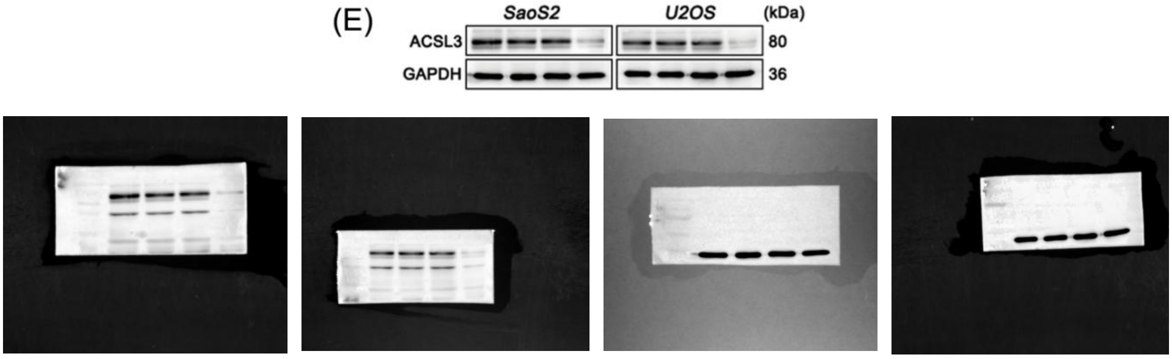

Fig. S6 Supplementary information of western blots in Fig. 5E.

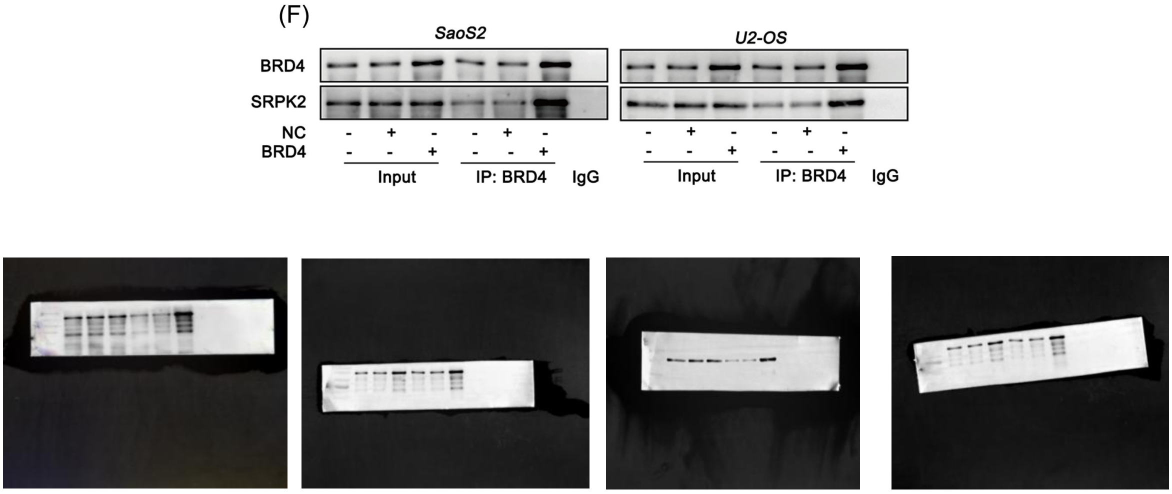

Fig. S7 Supplementary information of western blots in Fig. 6F.

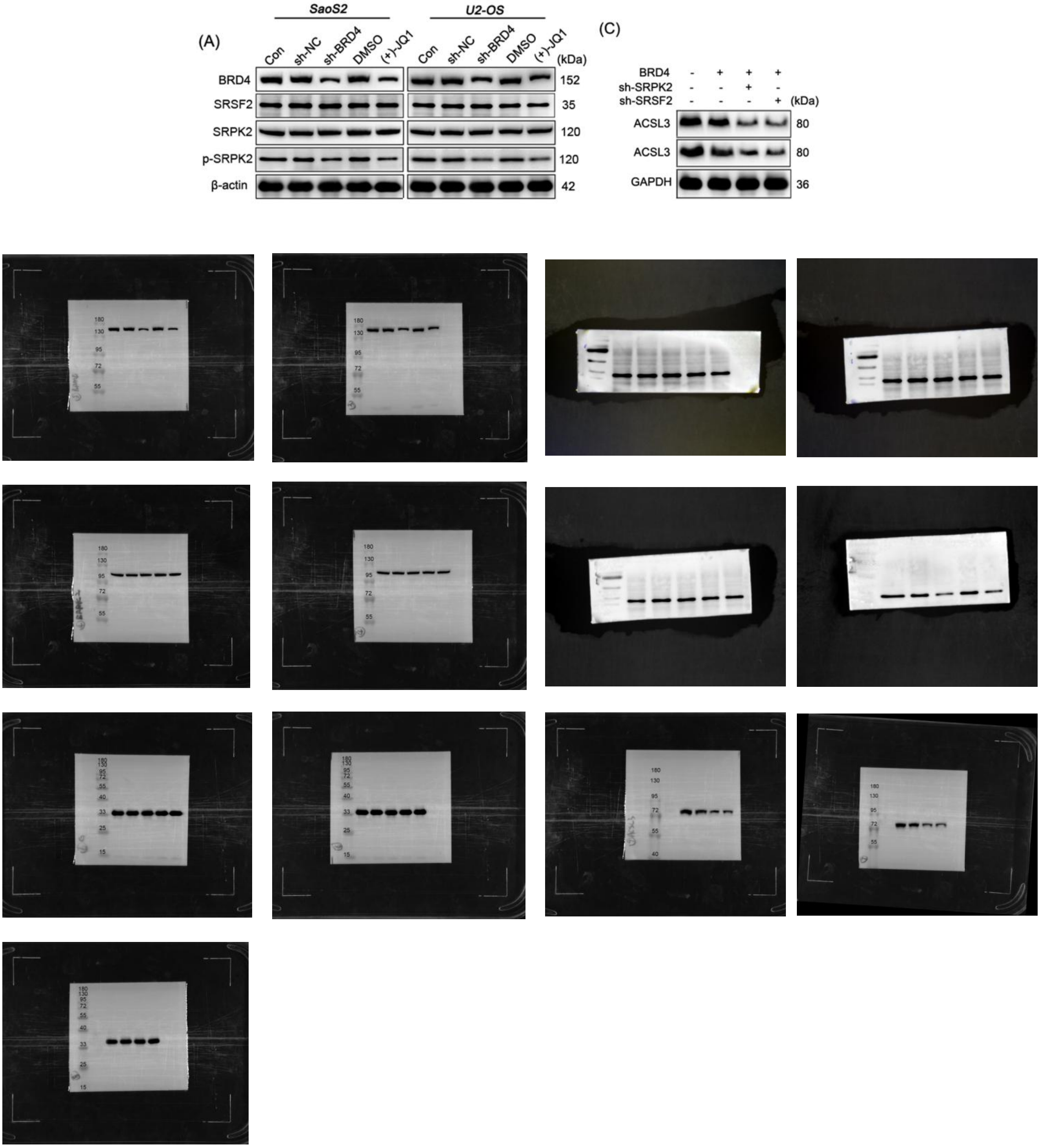

Fig. S8 Supplementary information of western blots in Fig. 7A & 7C.

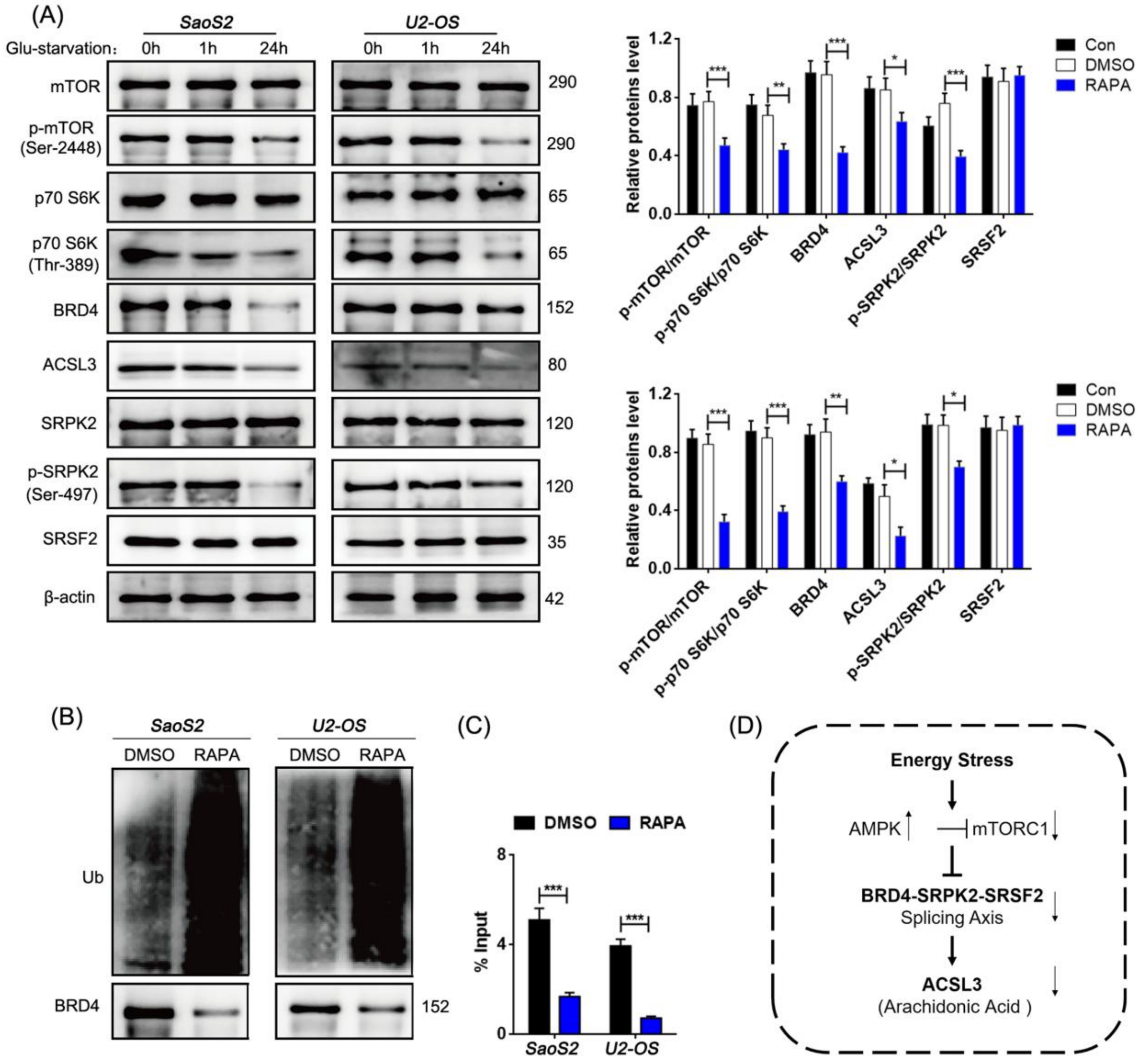

Fig.S9 Glucose-starvation induced mTOR signaling pathway significantly affects the BRD4-SRPK2-SRSF2 splicing axis. (A) Relative protein expression detected by western blotting. (B) Detection of ubiquitination level of BRD4 when mTOR was inhibited by rapamycin. (C) Pre-mACSL3 binding on SRSF2, displayed in percentage of the Input (% Input). (D) Schematic diagram of pre-ACSL3 splicing regulation in cells under energy stress.
